# Analysis of Risk Factors and Long-Term Outcomes in Kidney Transplant Patients with Identified Lymphoceles

**DOI:** 10.3390/jcm9092841

**Published:** 2020-09-02

**Authors:** Lukas J. Lehner, Arnim Hohberger, Lisanne Marschke, Nils Lachmann, Robert Peters, Frank Friedersdorff, Dmytro Khadzhynov, Fabian Halleck, Klemens Budde, Oliver Staeck, Michael Duerr

**Affiliations:** 1 Department of Nephrology and Intensive Care Medicine, Charité Universitätsmedizin Berlin, 10115 Berlin, Germany; arnim.hohberger@gmx.net (A.H.); lisanne.marschke@gmx.de (L.M.); dmytro.khadzhynov@charite.de (D.K.); fabian.halleck@charite.de (F.H.); klemens.budde@charite.de (K.B.); michael.duerr@charite.de (M.D.); 2 Center for Tumor Medicine, H&I Laboratory, Charité Universitätsmedizin Berlin, 13353 Berlin, Germany; nils.lachmann@charite.de; 3 Department of Urology, Charité Universitätsmedizin Berlin, 10115 Berlin, Germany; robert.peters@charite.de (R.P.); frank.friedersdorff@charite.de (F.F.); 4 KfH Kuratorium für Dialyse und Nierentransplantation e.V., 10559 Berlin, Germany; oliver.staeck@charite.de

**Keywords:** kidney transplantation, adverse events, lymphoceles, donor specific antibodies, allograft rejection

## Abstract

The collection of lymphatic fluids (lymphoceles) is a frequent adverse event following renal transplantation. A variety of surgical and medical factors has been linked to this entity, but reliable data on risk factors and long-term outcomes are lacking. This retrospective single-center study included 867 adult transplant recipients who received a kidney transplantation from 2006 to 2015. We evaluated for patient and graft survival, rejection episodes, or detectable donor-specific antibodies (dnDSA) in patients with identified lymphoceles in comparison to controls. We identified 305/867 (35.2%) patients with lymphocele formation, of whom 72/867 (8.3%) needed intervention. Multivariate analysis identified rejection episode as an independent risk factor (OR 1.61, CI 95% 1.17–2.21, *p* = 0.003) for lymphocele formation, while delayed graft function was independently associated with symptomatic lymphoceles (OR 1.9, CI 95% 1.16–3.12, *p* = 0.011). Interestingly, there was no difference in detectable dnDSA between groups with a similar graft and patient survival in all groups after 10 years. Lymphoceles frequently occur after transplantation and were found to be independently associated with rejection episodes, while symptomatic lymphoceles were associated with delayed graft function in our cohort. As both are inflammatory processes, they might play a causative role in the formation of lymphoceles. However, development or intervention of lymphoceles did not lead to impaired graft survival in the long-term.

## 1. Introduction

Perirenal fluid collections of various sizes and locations are a common complication after kidney transplantation [1,2]. Lymphocele formation is the most frequent complication and can be differentiated from hematoma, seroma, urinoma, and more rare causes by imaging (e.g., ultrasound or computed tomography), the course over time, or ultimately by fluid sampling and laboratory diagnosis [2,3,4,5]. Lymphoceles are commonly defined as a perirenal accumulation of lymph fluid surrounded by pseudo-membranes and may originate from the recipient´s lymphatics at the wound site or from the renal allograft [2,6,7]. The majority of lymphoceles occur in the first year after transplantation with an incidence ranging in between 0.6% to 33.9% of patients, depending on surgical technique, medical factors, and the intensity of imaging post-transplant [2]. However, lymphocele formation has been reported up to 3.7 years post-transplant [8]. Although the majority of lymphoceles remain asymptomatic, it is of crucial importance to detect lymphoceles early in the course, since complications are usually severe, including compression of adjacent vessels, deep vein thrombosis, edema of the ipsilateral limb, abdominal discomfort, urinary obstruction, and infection. This may lead to severe impairment of the graft function or even graft loss. In such cases a timely assessment and minimal invasive or even open surgical intervention is required to avoid further deterioration [1,2,9,10,11]. A variety of surgical and medical risk factors has been linked to this entity. Surgical causes comprise dissection of lymphatic tissue around the iliac vessels of the recipient and dissection of the recipient’s lymphatics during procurement or preparation of the organ prior to re-implantation [2]. In addition, medical factors such as diabetes [10], high body mass index (BMI), delayed graft function (DGF) [12], use of mammalian target of rapamycin (mTOR) inhibitors [1,13,14,15] and acute rejection have been associated with lymphocele formation [1,10,16]. Rashid et al. described an association of lymphoceles with rejection in 1974 [17]. Subsequently, a significant risk for the development of lymphoceles following acute rejection was found in various multivariate analyses [1,2,8]. Furthermore, pathological studies of several groups revealed important insights into the pathophysiological aspects of donor-related lymphocele formation by rejection, and cellular infiltrates within the graft and their association with lymph angiogenesis [2,18,19,20,21,22,23].

Acute rejection has been linked with the development of donor-specific antibodies (DSA) [24,25], potentially leading to acute or late antibody-mediated rejection [26]. ABMR has been shown to have detrimental effects on graft function [27,28], either in the short or long-term, ultimately leading to graft failure, while treatment remains challenging [26].

Data on long-term outcomes on graft and patient survival with additional information on immunological aspects such as the evolution of DSA in the context of lymphocele formation are scarce. In the present study, we aimed to identify the incidence and clinical course of this complication, as well as long-term outcome in a well described cohort of renal transplant patients and adequate controls. 

## 2. Experimental Section

### 2.1. Patient Selection and Data Acquisition

In the present retrospective single-center study, we included all 873 adult patients who received a donation after brain death (DBD), or living donor kidney transplantation, between 1st January 2006 and 31st December 2015 at the transplant center Charité Campus Mitte in Berlin. All patients were routinely screened by regular repeated graft sonography in the post-transplantation period. An experienced physician investigated patients with standardized ultrasound procedures. Routine sonography is performed daily in the first week after transplantation, two-times times per week within the first month, one-two times/month to the end of month 6, and on indication after that. Thereby, subjects with classical features of lymphocele formation (perirenal anechoic pseudo-cystic structures with or without septal internal structures) as described by the sonographer were identified. Patients with fluid collections resembling hematoma or wound-site associated seroma, as well as patients with proven urinoma (*n* = 0), were excluded from the analysis. In the further analysis of outcome parameters, we subdivided the lymphocele cohort based on clinical management into two different groups. Cases with conservative lymphocele management were assigned to group A (conservative), and patients with a history of any interventional lymphocele treatment were assigned to group B (therapy). Other patients with no evidence of lymphoceles were control patients, group C.

We followed the transplant outcome till 31st December 2016. For data collection, our electronic patient database “TBase” [29] and clinical charts yielding complete data sets were used. The institutional ethics committee (EA 1/048/14) approved all study procedures.

### 2.2. Definitions

We used the necessity of dialysis within the first week post-transplant as the definition of delayed graft function (DGF). For calculation of kidney donor profile indices (KDPI) for deceased donors, the Organ Procurement and Transplantation Network (OPTN) calculation rules and mapping table referring to the median donor of 2014 as reference was used [30,31]. The living kidney donor profile indices (LKDPI) for living donors were calculated according to the methods described by Massie et al. [32].

### 2.3. Rejection Episodes

A rejection episode was defined by either borderline rejection or T-cell mediated rejection (≥BANFF Ia) or antibody mediated rejection on biopsy, according to the BANFF 2013 criteria [33]. At our center, re-transplantation underlies a strict policy of defining all antigens from previous transplantations as forbidden antigens, leading to a very low incidence of early antibody mediated rejection (ABMR) in the first year. As ABMRs always presented in combination with a cell mediated component, no isolated ABMRs were captured. We performed ultrasound workup in all patients, including a comprehensive status of the renal graft regarding size, parenchymal thickness, perfusion, and signs for postrenal obstruction, prior to ultrasound guided biopsies.

### 2.4. Immunosuppression

Most patients in the study period initially received a standard immunosuppressive protocol, including induction therapy with an anti-IL2-R antibody, a calcineurin inhibitor, mycophenolate, and steroids. A minority of patients received “other” immunosuppression within clinical trials for approval of new therapies or treatment regimes. Tapering of steroids was performed to achieve a steroid-free regimen after the first year if no rejection episodes had occurred.

### 2.5. Evaluation of De Novo Donor-Specific Antibodies

Transplants were performed based on negative complement-dependent cytotoxicity (CDC) crossmatch using isolated T- and B-lymphocytes. Pre-transplant human leucocyte antigen (HLA)-antibodies were determined by CDC in combination with solid-phase immunoassays. All patients on the kidney waiting list were tested by the Luminex®-based LABScreen® mixed and single antigen bead (SAB) assay (One Lambda, Canoga Park, CA). A cross-sectional post-transplant HLA-antibody monitoring scheme by SAB, including all kidney transplants with a functioning allograft, was performed annually, and in case of clinical signs of impaired allograft function [34]. All tests were performed according to the manufacturer’s instructions. For the SAB assay, a normalized mean fluorescence intensity value exceeding 1000 was defined as positive in the pre- and post-transplant setting. The HLA loci A, B, C, DRB, and DQB were considered for the definition of de-novo donor-specific antibodies (dnDSA) [34].

### 2.6. Surgical Technique and Postoperative Treatment

A para-rectal incision using the standard extraperitoneal technique was performed to place the graft in the iliac fossa. In primary transplantation and especially in re-transplanted patients the choice of site was usually preplanned by an interdisciplinary team (including transplant-urologists, radiologists, nephrologists and vascular surgeons) based on the anatomic situation. The allograft artery was anastomosed in a side-to-end and the renal vein in an end-to-side fashion to the external iliac vessels of the recipient. Thereby, our experienced surgical team (>80 transplants/year) meticulously tried to spare the iliac structures of the patients and the graft tissue with diligent ligation of open lymph vessels to minimize lymphorrhea. After vesicoureteral anastomosis in Lich-Gregoir or full-thickness technique with two running sutures, the ureter was stented routinely by a double J-catheter. After renal transplant procedure the Robinson drainage system was removed as soon as possible when the drainage volume was below 50 mL/day. In all patients the double J-catheter remained for three-six weeks to prevent ureteral obstruction. Treatment of symptomatic lymphoceles was performed by either incision with percutaneous drainage, laparoscopic fenestration or open surgery. Of note, sclerotherapy of lymphoceles was not performed at our center. The choice of treatment was made on an interdisciplinary case by case decision depending on the anatomic situation and was guided by the current guidelines.

### 2.7. Data Analysis

Donor and recipient characteristics are shown as means with standard deviation or in case of non-normal distribution as median with interquartile range (IQR of 25th and 75th percentile). Data were analyzed by the chi-square test, Fisher exact, ANOVA and t-tests for independent variables. In the case of a non-parametric distribution, the Kruskall-Wallis test was used. The significance level was set to α = 0.05. Kaplan-Meier and logistic as well as Cox regression analyses were performed to determine predictors for lymphocele formation, graft and patient survival with a confidence interval of 95%. No missing values were substituted, due to a complete data set. For the multivariate analysis, a stepwise backward elimination with inclusion of recipient age, donor age, living donation, cold ischemia time, delayed graft function (DGF), number of HLA mismatches, re-transplantation, recipient diabetes, BMI, rejection episode, lymphocele formation and lymphocele treatment where applicable was used. IBM (IBM Germany GmbH, IBM-Allee 1, 71139 Ehningen, Germany) SPSS 25 for mac was used for statistical analysis. 

## 3. Results

### 3.1. Patient characteristics

In the period between 2006–2015, we identified 867 adult transplant recipients who received a kidney from either deceased or living donors. The overall incidence of lymphocele formation was 305 out of 867 patients (35.2%), of which only 72 (8.3%) required treatment for symptomatic lymphoceles. We subclassified patients into conservative (group A; *n* = 233), interventional treatment (group B; *n* = 72), and control group (group C; *n* = 562). All data on patient demographics and clinical characteristics are displayed in Table 1. In all three patient groups, we found no significant difference concerning gender, re-transplantation, time on dialysis before transplantation, HLA mismatches, body mass index, diabetes rate, and the living donor quality index - LKDPI. Of note, patient age and donor age were higher within the lymphocele groups (group A + group B) compared to the control group. Median cold ischemia time (CIT) was similar in all groups (overall cadaveric donations: 11.2 (IQR 8–14.8) hours and overall living donation 2.5 (IQR 2.2–3.1) hours). In the control group, the proportion of living donation was significantly higher compared to group A and B (37% vs. 26% vs. 19%, *p* < 0.001). The deceased kidney donor quality index (KDPI) was highest in the lymphocele treatment group (group B). The mean follow-up time of the entire cohort was 5.7 (SD 2.8) years. 

A higher proportion of patients in the lymphocele groups received cyclosporine-based therapy, whereas the majority of control patients were treated with tacrolimus. Figure S1 demonstrates the proportion of ciclosporin A and tacrolimus treated patients over the years. At transplantation, only ten patients received a regime comprising mTOR inhibition within interventional studies. These patients were evenly distributed between the analyzed groups. The use of mycophenolic acid and other immunosuppressants was similar between groups.

### 3.2. Course and Treatment Of Lymphoceles

**Figure 1:**
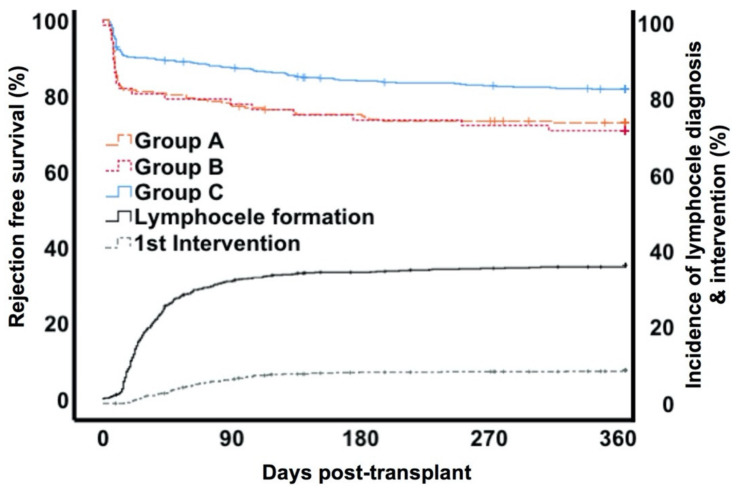
Timing of rejection episodes, first diagnosis of lymphocele formation and first intervention of lymphoceles. * Group A= lymphocele conservatively monitored; Group B= lymphocele with intervention; Group C= control.

**Figure 2:**
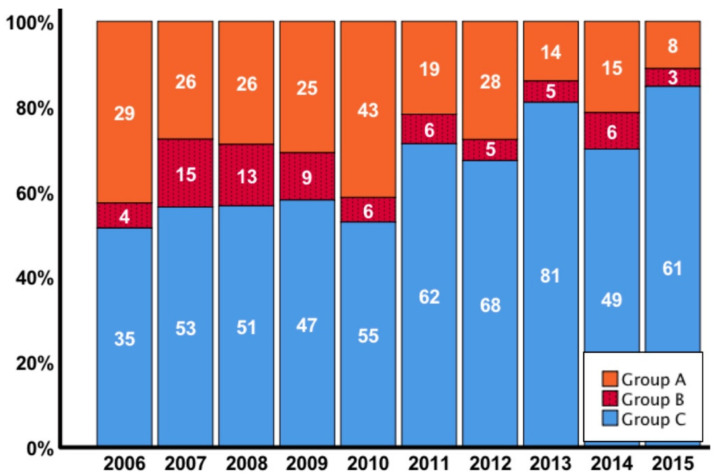
Proportion and absolute numbers of lymphocele formation, intervention and control by year. * Group A= lymphocele conservatively monitored; Group B= lymphocele with intervention; Group C= control.

The median time of diagnosis of lymphocele formation was 29 days (IQR 19–51) after transplantation. Patients in whom a lymphocele developed underwent repetitive ultrasound tests to detect possible complications. In the majority of patients (76%, *n* = 233) fluid collections resolved spontaneously over time. Symptomatic lymphoceles occurred in 72 patients (group B) requiring interventional treatment, which was performed in the median 22 days (IQR 8–55) after diagnosis (Figure 1). Of these patients 13/72 (18.1%) demonstrated an acute kidney injury (AKI grade I-II as classified by the Kidney Disease Improving Global Outcomes (KDIGO) guidelines 2012) at this point [35]. The majority of interventions were performed laparoscopically (*n* = 61 (84.7%). Incision with drainage was performed in seven cases (9.7%) and four (5.6%) patients had to be treated with open surgery. We found 18 cases suffering from a relapse of the lymphocele (13 (22%) patients in the laparoscopic fenestration, 4 (40%) patients in the drainage, and 1 (33%) patient in the open surgery group). Patients with relapse of lymphoceles were primarily treated by laparoscopic fenestration (14 cases), while four patients needed open surgery. The third intervention by either laparoscopic fenestration or open surgery was necessary for two patients. Regarding complications after laparoscopic fenestration, five patients suffered from injuries of the urinary bladder or the ureter. All post-surgery complications are summarized in Table S1. In the end, all cases could be treated successfully. Figure 2 demonstrates symptomatic and asymptomatic lymphocele formation in comparison to control by transplant year. 

### 3.3. Rejection and Graft Function

Over the observation time, biopsy proven rejection episodes (including borderline, antibody mediated rejections and T-cell mediated rejection) occurred in group A in 72/233 (30.9%) patients, in group B in 25/72 (34.7%) and in group C 122/562 (21.7%) (*p* = 0.004). Out of these, “borderline rejection” was found in 24/72 (33.3%, group A), 7/25 (28%, group B) and 30/122 (24.5%, group C) patients. T-cell mediated rejection rates were 20.6% (48/233) in group A, 25% (18/72) in group B and 16.3% (92/562) in group C patients (*p* = 0.111). All groups showed a similar distribution in the BANFF I-III classifications (*p* = 0.406). In the first year, the proportion of mixed rejections in terms of the combination of cell-mediated rejection and antibody mediated rejection (ABMR) was 2.1% (5/233) patients in group A, 1.3% (1/72) in group B and 0.2% (14/562) in group C (*p* = 0.827). In our cohort we did not find any isolated ABMR. Within group A 46 patients developed lymphoceles on average 27 (IQR 17–43) days after the rejection episode. In 26 patients the rejection episode occurred 164 (IQR 75–463) days after the first diagnosis of a lymphocele. Within group B 15 patients had lymphoceles diagnosed 31 (IQR 16–57) days after the rejection episode. In the other remaining 10 patients rejection episodes occurred 277 (IQR 75–464) days after the first diagnosis of a lymphocele and 240 (IQR 94–491) days after intervention. The time course of lymphocele development, intervention and rejections is demonstrated in Figure 1. Importantly, in patients treated with cyclosporine the rejection rates within the first year post-transplant were significantly higher (CyA: 26.7% (CI 95% 24.5–28.9)) than in the tacrolimus treatment group (tacrolimus: 16.6% (CI 95% 14.8–18.4); *p* = 0.001) (Supplementary Figure S2).

Delayed graft function was more frequent in both lymphocele groups (42.1% vs. 52.7% vs. 32.4%, *p* < 0.001); however, we did not find a significant difference in the creatinine nadir after transplantation (group A: 1.1 (IQR 0.9–1.4) mg/dL vs. group B: 1.2 (IQR 1–1.5) mg/dL vs. group C 1.1 (IQR 0.9–1.4) mg/dL, *p* = 0.113).

### 3.4. Risk Factors for Lymphocele Formation and Treatment

Risk factors for lymphocele formation and treatment were analyzed in univariate and multivariate analyses (Table 2). On multivariate analysis living donation was associated with a lower risk (odds ratio (OR) 0.55, CI 95% 0.4–0.75, *p* < 0.001), whereas patients with a rejection episode had a 1.61 fold higher risk (CI 95% 1.17–2.21, *p* = 0.003) for lymphocele formation. However, in the multivariate analysis of transplant recipients with lymphoceles that needed intervention (group B) mean donor age (OR 1.02, CI 95% 1–1.04, *p* = 0.023) and delayed graft function (OR 1.9, CI 95% 1.16–3.12, *p* = 0.011), but not rejection episode, showed a significant association. Moreover, overall lymphocele formation was significantly higher in the Ciclosporin A group within the first year post-transplant (CyA 42.9 (CI 95% 40.4–45.4) vs. tacrolimus 27.9% (CI 95% 25.7–30.1); *p* < 0.001) (Figure S3).

### 3.5. Donor Specific Antibody Formation

**Figure 3:**
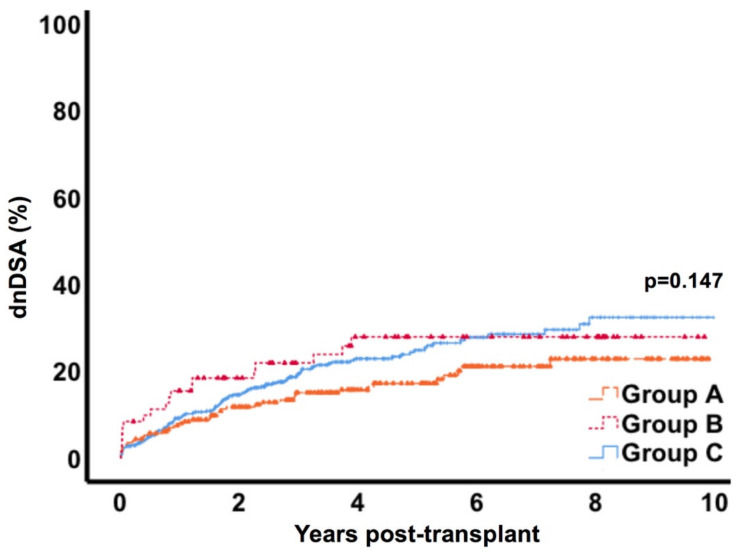
Formation of donor-specific antibodies post-transplant. * Group A= lymphocele conservatively monitored; Group B= lymphocele with intervention; Group C= control.

Formation of de novo donor specific antibodies (dnDSA) occurred in 40/233 (17.2%) patients in group A, in 18/72 (25%) in the group B and in 115/562 (20.5%) patients in group C. Figure 3 displays dnDSA formation over a 10-year follow-up and shows similar rates in all groups. Multivariate analysis confirmed risk factors for dnDSA formation, with significant associations for recipient age (hazard ratio (HR) 0.98, CI 95% 0.97–0.99, *p* = 0.001), living donation (HR 0.58, CI 95% 0.4–0.84, *p* = 0.004), re-transplantation (HR 1.78, C I95% 1.16–2.74, *p* = 0.008) and HLA-mismatches (HR 1.43, CI 95% 1.28–1.6, *p* < 0.001), but not for lymphocele formation (HR 0.79, CI 95% 0.57–1.1, *p* = 0.161) in particular.

### 3.6. Patient and Graft Survival

**Figure 4:**
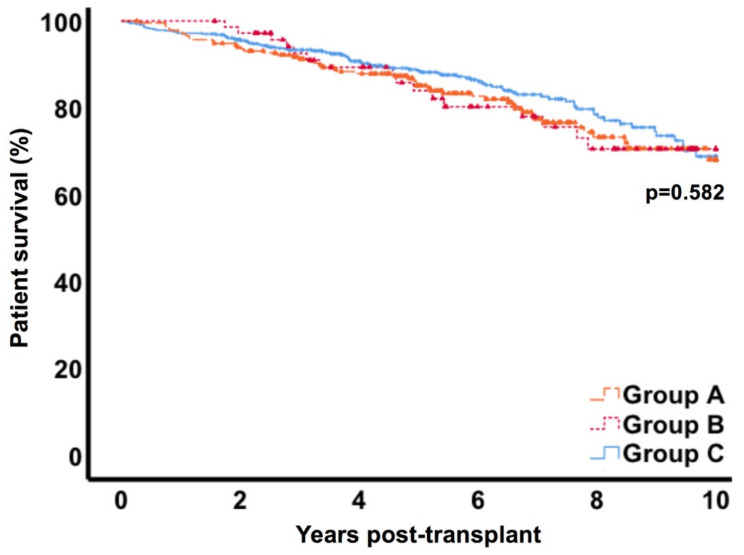
Patient survival. * Group A= lymphocele conservatively monitored; Group B= lymphocele with intervention; Group C= control.

**Figure 5:**
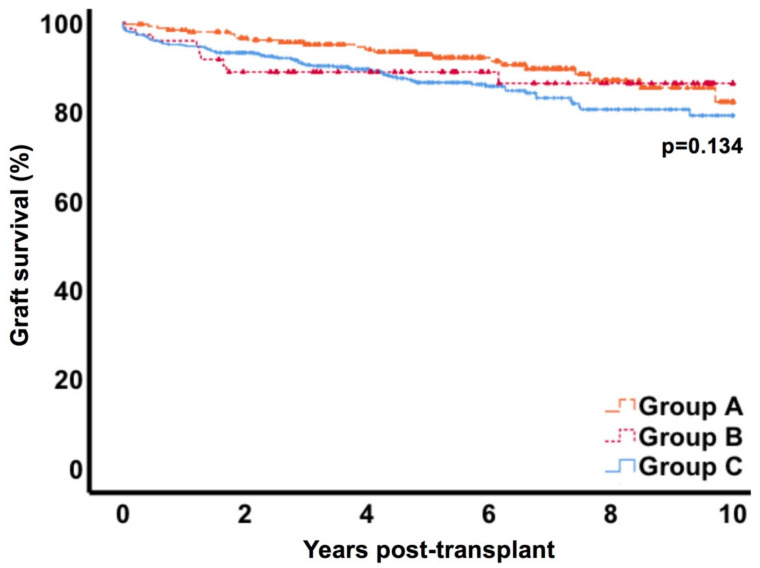
Death censored graft survival. * Group A= lymphocele conservatively monitored; Group B= lymphocele with intervention; Group C= control.

After 10 years, overall patient survival was 68.1% (CI 95% 63.6–72.6) in group A, 70.5% (CI 95% 64.1–76.9) in group B and 68.8% (CI 95% 65.1–72.5) in control C (*p* = 0.582) (Figure 4). Death-censored graft survival at 10 years was similar in the three groups with 82.2% (CI 95% 77.8–86.6), 86.3% (CI 95% 81.9–90.7) and 79.1% (CI 95% 76.4–81.8) respectively (*p* = 0.134) (Figure 5). Uni- and multivariate analysis for risk factors of graft loss in our cohort revealed donor age, re-transplantation, delayed graft function and rejection episode, but not lymphocele treatment, as being associated (Table 3). Besides, within the workup of cases in group B we did not find any graft loss directly attributable to symptomatic lymphoceles. 

## 4. Discussion

Lymphoceles, in terms of a pseudocyst filled with lymphatic fluids around the kidney allograft [6,7], may originate from the recipient iliacal lymphatics or the renal graft [2,14]. The incidence of this surgical complication has been described in 0.6 to 33.9% of patients [2,6,7,10,36,37]. Incidence rates vary widely in the literature due to different definitions referring to size and different diagnostic regimes. In our cohort, we found 35% of 867 patients diagnosed with lymphocele formation, primarily as an incidental finding on routine ultrasound examination. This number appears to be slightly higher than previously described. However, abdominal ultrasound follow-up is performed frequently in the post-operative period at our center and we did not use a cut-off concerning the extent of the fluid collections. Routine sonography is performed daily in the first week after transplantation, two-three times a week within the first month, one-two times/month to the end of month 6 and half-yearly or on indication after that. Despite the relatively high incidence of lymphocele formation, the rate of symptomatic lymphoceles necessitating treatment was low (only 8.3% of 867 patients), and relapses after treatment were rare (2%) and successfully treated in all cases without any associated graft loss. In our study we used a rigorous ultrasound (US) follow-up protocol, which might explain the rather high rate of asymptomatic lymphoceles. Currently, there is no evidence for such a close US scheme, which results in higher costs, without clear evidence for added benefits in hard outcomes. The availability of an adequately equipped and staffed US department enabled us to closely monitor the patients and properly describe all relevant US abnormalities with high accuracy for this research project.

Of medical risk factors, the association of rejections with lymphocele formation has already been reported in various studies [1,10,16,17,38]. In our cohort, multivariate analysis revealed rejection episodes and delayed graft function as the strongest independent risk factors for development of lymphoceles. In symptomatic lymphoceles only DGF remained significant on multivariate testing, despite a significantly higher rate of rejection episodes compared to control. Of note, we also found higher rates of rejection in cyclosporine treated patients (Figure S2), who, in addition, demonstrated higher rates of lymphocele formation in the first year post-transplant (Figure S3). This effect might in part explain the notably higher incidence of lymphocele formation in the years 2006–2010, since in the following years the proportional use of tacrolimus increased markedly (Figure 2 and Figure S1). Besides, in these years the surgical team also changed and from continuous evaluation of complications an increased focus on sclerosing and clipping of injured lymphatic tissue evolved. For the multivariate analysis, “borderline”, rejections according to Banff criteria (≥BANFF Ia) and mixed rejections due to simultaneously occurring early antibody mediated rejections were combined since these entities resemble significant inflammation in the transplanted kidney, which has been associated with lymph angiogenesis in renal allografts and subsequently to lymphocele formation [19,21,22,23,39]. The potential underlying mechanism remained elusive, until Kerjaschki and his group were able to demonstrate increased lymph angiogenesis, augmented 50-fold in rejection associated inflammatory infiltrates [21]. However, in our analysis biopsy-proven acute rejections excluding the borderline readings did not demonstrate significant differences between groups, as well as the BANFF gradings I-III. This finding argues against an inflammation-grade dependent mechanism. Similar results were reported by two other groups [23]. In our cohort we were not able to draw a direct correlation of clinical with histologic results, since our nephropathologist only reported biopsy readings based on BANFF classification in the study period. Staining for lymphatics and readings of lymph vessel status or on lymph angiogenesis were not covered. However, Stuht and his group did not find a correlation with acute rejection but found a higher density of lymphatics in the areas with cellular infiltrates irrespective of their origin [22]. Delayed graft function has been associated with lymphocele formation in a study by Khauli et al. in univariate analysis, but this was not confirmed in multivariate testing [8]. Our cohort matches these findings, but in addition, we found a correlation with DGF and symptomatic lymphoceles in the multivariate analysis. Both lymphocele groups received grafts with significantly higher KDPI values, and particularly group B patients received kidneys from patients with higher age. These are indicators for poorer quality kidneys and, together with a lower proportion of living donation, might explain these findings. Nevertheless, DGF has been linked with inflammation, partly because of reperfusion injury [40,41], a fact that might also play a role.

Due to the association of lymphocele formation with rejection episodes and inflammatory conditions, we expected potential formation of de novo donor specific antibodies (dnDSA) in this context. Moreover, in theory, increased contact of lymphatic fluid containing cell constituents could further contribute to dnDSA development. Therefore, we analyzed dnDSA formation up to 10 years after transplantation and found similar rates in the three groups. In addition, lymphocele formation was not attributable to dnDSA development on multivariate testing.

In general, data on long-term outcomes after formation of asymptomatic or symptomatic lymphoceles are scarce. In our cohort, we found similar rates of patient survival in the lymphocele groups compared to the control group. Also, death-censored graft survival rates did not differ significantly after 10 years in all groups with 82.2% (CI 95% 77.8–86.6) in group A, 86.3% (CI 95% 81.9–90.7) in group B and 79.1% (CI 95% 76.4–81.8) in group C (*p* = 0.134). Our findings are in accordance with the studies of Zagdoun et al. and Smyth et al., who found comparable graft survival rates over six years post-transplant [11] and a mean follow-up of 63 months [42]. Veeramani et al. also report similar graft survival rates with lymphoceles and the control group. However, this cohort consisted primarily of living transplants, since only nine patients of 1709 received a cadaveric allograft [16].

Although follow-up is complete and long, this retrospective analysis had certain limitations: different group sizes and low number of patients in specific subgroups, low patient numbers with preformed DSA, limited data on any wound complications or dataset on albumin status, limited data on re-operations after transplantation, insufficient data on warm ischemia time during transplant procedure, and low patient numbers on mTOR inhibitors.

The strong association of mTOR inhibitors with lymphocele formation and its wound healing effects have been described by several groups [1,13,15] and was already observed in our center in 2003 [14]. In spite of this, our data are in line with data from Germany in 2012 (1,6% mTOR in de novo patients [43]) and from the U.S. over recent years [44].

Regarding detection of persistent lymphorrhea we found 25% patients needed re-operation due to recurrent lymphocele formation, which, fortunately, could be treated successfully in this cohort. Based on all available data, persistent lymphorrhea was not seen in our cohort, with the limitation that duration and daily amount of lymph fluid production were not documented in our database in this retrospective study.

Our study did not find an association for dnDSA and lymphocele formation, but the small sample size and low frequency of humoral rejections may limit our conclusion. To address this limitation, we suggest performing a prospective study with quarterly HLA-testing, rigorous documentation of clinical events, combined with routine ultrasound follow-ups with above described intervals, or at any re-hospitalization. With adequate patient numbers based on a power analysis of our presented data, this prospective study could provide robust data in this regard.

In conclusion, lymphoceles frequently occur after transplantation and were found to be independently associated with rejection episodes, while symptomatic lymphoceles were associated with delayed graft function in our cohort. Although both are inflammatory processes and might play a causing role in the formation of lymphoceles, this did not add significant risk for dnDSA development. Moreover, development or intervention of lymphoceles did not lead to compromised graft survival in the long-term.

### Considerations for Clinical Practice

In order to detect complications (such as lymphoceles, hematoma, vascular problems or irregular perfusion) early, we believe that close ultrasound monitoring until discharge is important, e.g., every two or three days immediately after transplantation, after the removal of the bladder catheter or the ureter stent, as well as in case of any problems. In the outpatient setting, regular ultrasound investigations should be scheduled depending on availability in the center, ideally at least once in the first month, and once until month 6. At annual follow-up visits the ultrasound investigation should also include the native kidneys, as renal cell carcinoma may develop. In case of complications and deterioration of graft function, an ultrasound investigation is mandatory. Unfortunately, there is no good scientific evidence for such a recommendation. In order to generate good evidence more studies are needed and ultimately a prospective randomized study to compare outcome and costs of a tight regular ultrasound monitoring schedule in comparison to a less rigorous approach. Such a prospective study could address the present limitations of our study. Patients should always be asked about and investigated for any signs of limb discomfort as a correlate for compression of inguinal vessels due to a putative lymphocele formation. In the case of suspicious lymphocele formation, findings should be discussed interdisciplinarily in the context of graft function, vascular complications or signs of infection to plan the best procedure for the individual patients according to the general treatment recommendations of the European Association of Urology [45].

## Figures and Tables

**Table 1:** Patient characteristics of subjects with conservatively monitored lymphoceles (group A), lymphocele treatment (group B) and of the control group (group C).

	Group A	Group B	Group C	*p*-Value
*n* (number), (%)	233 (26.8%)	72 (8.3%)	562 (64.8%)	
Mean follow up, years (SD)	6.2 (2.8)	6.5 (2.8)	5.4 (2.8)	0.006
Mean recipient age, years (SD)	52 (14)	54 (15)	50 (15)	0.016
Mean donor age, years (SD)	53 (15)	58 (13)	53 (15)	0.027
Male, *n*	149 (64%)	40 (56%)	335 (60%)	0.339
Re-transplantation, *n*	21 (9%)	12 (17%)	60 (11%)	0.189
Median time on dialysis, months (IQR)	53 (21–87)	61 (22–85)	47 (12–84)	0.112
Dialysis modality, *n*				0.09
Hemodialysis:	201 (86.3%)	61 (84.7%)	436 (77.6%)	
Peritoneal dialysis:	14 (6%)	7 (9.7%)	47 (8.4%)	
Preemptive:	10 (4.3%)	2 (2.8%)	44 (7.8%)	
Unknown:	8 (3.4%)	2 (2.8%)	35 (6.2%)	
Mean HLA-A, B, DR mismatches (SD)	2.8 (1.7)	2.9 (1.5)	2.8 (1.7)	0.906
Median cold ischemia time (cadaveric donation), hours (IQR)	11 (8.1–15.2)	11.1 (7.5–15.5)	11.2 (8–14.4)	0.961
Median cold ischemia time (living donation), hours (IQR)	2.5 (2.2–3)	2.4 (2.1–3.2)	2.5 (2.2–3.1)	0.99
Body mass index, kg/m^2^ (SD)	25.8 (4.5)	25.6 (4.3)	25.8 (4.6)	0.939
Pre-Tx Diabetes (all types)	22 (9.4%)	8 (11.1%)	42 (7.5%)	0.391
Living donation, *n*	60 (26%)	14 (19%)	210 (37%)	<0.001
**Donor creatinine, mg/dL (IQR)**	0.86 (0.70–1.04)	0.87 (0.71–1.11)	0.80 (0.69–0.99)	0.103
KDPI (SD)(deceased donor kidneys, *n* = 581)	65 (29)	77 (23)	63 (30)	0.004
Living kidney donor profile indices (LKDPI) (living donor kidneys, *n* = 284)	20 (24)	20 (29)	23 (24)	0.572
Delayed graft function, *n*	98 (42.1%)	38 (52.7%)	182 (32.4%)	<0.001
**Preformed donor specific antibodies**, *n*	6 (0.7%)	1 (0.1%)	27 (3.1%)	0.173
Rejection episode (T-cell mediated rejection + antibody mediated rejection + Borderline)	72 (30.9%)	25 (34.7%)	122 (21.7%)	0.004
T-cell mediated rejection	48 (20.6%)	18 (25%)	92 (16.4%)	0.111
Primary immunosuppression at transplantation				
- Tacrolimus	99 (43%)	22 (31%)	314 (56%)	<0.001
- Cyclosporine	127 (55%)	47 (65%)	233 (42%)	<0.001
- mammalian target of rapamycin (mTOR) inhibitor	4 (2%)	1 (1%)	5 (1%)	0.499
- mycophenolic acid	224 (96%)	71 (99%)	542 (96%)	0.699
- others	19 (8%)	3 (4%)	37 (7%)	0.523
- Basiliximab induction	all patients			

**Table 2:** Risk factors for lymphocele formation (group A) and treatment (group B) using a logistic regression model.

	Group A				Group B			
	Monovariate		Multivariate		Monovariate		Multivariate *	
Variable	OR	*p*-Value	OR	*p*-Value	OR	*p*-Value	OR	*p*-Value
Mean recipient age per year	1.01	0.008			1.02	0.039		
Mean donor age per year	1.01	0.131			1.02	0.008	1.02	0.023
Male sex	0.91	0.498			1.25	0.377		
Re-transplantation	1.02	0.948			1.76	0.093		
Median time on dialysis per month	1.00	0.079			1.00	0.568		
Mean HLA-mismatches per mismatch	1.01	0.835			1.03	0.657		
Median cold ischemia time per hour	1.03	0.005			1.04	0.035		
Donor creatinine per mg/dL	1.15	0.189			1.15	0.373		
Living donation	0.54	<0.001	0.55	<0.001	0.47	0.014		
BMI per kg/m^2^	1.00	0.779			0.99	0.755		
Diabetes	1.35	0.23			1.43	0.37		
Rejection episode	1.68	0.001	1.61	0.003	1.65	0.056		
DGF	1.68	0.001			2.06	0.004	1.9	0.011

* multivariate analysis using stepwise backward elimination adjusting for age, donor age, living donation, cold ischemia time, rejection episode, delayed graft function (DGF).

**Table 3:** Risk factor analysis for graft loss using a Cox proportional hazard model.

	Monovariate		Multivariate *	
n	HR	*p*-Value	HR	*p*-Value
Mean recipient age per year	1.03	<0.001		
Mean donor age per year	1.04	<0.001	1.04	<0.001
Male sex	1.29	0.180		
Re-transplantation	1.59	0.079	1.74	0.046
Median time on dialysis per month	1.00	0.230		
Mean HLA-mismatches per mismatch	1.24	<0.001		
Median cold ischemia time per hour	1.03	0.056		
Living donation	0.47	0.002		
Diabetes	2.34	0.001		
BMI per kg/m2	1.06	0.006		
Rejection episode	2.5	<0.001	1.94	0.001
DGF	2.52	<0.001	1.73	0.008
Lymphocele treatment	0.91	0.788	n.a.	n.s.

* multivariate analysis using stepwise backward elimination adjusting for age, donor age, re-transplantation, mismatches, living donation, BMI, delayed graft function (DGF), rejection episode, lymphocele treatment.

## Supplementary Materials

The following are available online at https://www.mdpi.com/2077-0383/9/9/2841/s1.
