# Supplementary material for: Analysis of Risk Factors and Long-Term Outcomes in Kidney Transplant Patients with Identified Lymphoceles
Source: J Clin Med. 2020 Sep 2;9(9):2841. doi: 10.3390/jcm9092841 (PMC7563120; doi:10.3390/jcm9092841)
Supplement: Supplementary file 1 [file jcm-09-02841-s001.pdf]

**Supplementary table 1:.**

| Pat ID | Indication for lymphocele (LC) intervention | Primary procedure | Post-surgery complications | Kidney function in context of lymphocele intervention |
|---|---|---|---|---|
| 1 | Persistent lymphatic drainage | Laparoscopic fenestration | 2x relapse - 2nd laparoscopic fenestration and open surgery necessary | Postrenal acute kidney injury (AKI) I° with necessity of re-implantation of a double J catheter |
| 2 | Infection of LC and postrenal pelvic ectasia of the transplant | Laparoscopic fenestration | None | Stable |
| 3 | Infection of LC | Incision with drainage | None | Stable |
| 4 | Postrenal complication | Laparoscopic fenestration | None | AKI I° |
| 5 | Postrenal complication | Laparoscopic fenestration | Relapse - 2nd laparoscopic fenestration necessary | Postrenal AKI II° |
| 6 | Progressive LC | Laparoscopic fenestration | None | Stable |
| 7 | Infection of LC | Open surgery | Relapse - laparoscopic fenestration necessary | Stable |
| 8 | Progressive LC | Laparoscopic fenestration | None | Stable |
| 9 | Post-renal complication | Incision with drainage | Relapse - Laparoscopic fenestration necessary | Stable |
| 10 | Progressive LC | Laparoscopic fenestration | None | Stable |
| 11 | Progressive LC, edematous distal limb swelling | Laparoscopic fenestration | None | Stable |
| 12 | Postrenal complication | Laparoscopic fenestration | None | Postrenal AKI I° |
| 13 | Progressive LC | Laparoscopic fenestration | None | Stable |
| 14 | Progressive LC | Laparoscopic fenestration | None | Stable |
| 15 | Progressive LC | Laparoscopic fenestration | None | Stable |
| 16 | Infection of LC | Laparoscopic fenestration | None | Stable |
| 17 | Progressive LC | Laparoscopic fenestration | Relapse - open surgery necessary | AKI I° |
| 18 | Progressive LC, compression of adjacent vessel | Laparoscopic fenestration | Relapse - 2nd laparoscopic fenestration necessary | Stable |

| | | | | |
|---|---|---|---|---|
| 19 | Progressive LC, compression of adjacent vessel | Laparoscopic fenestration | Relapse - 2nd laparoscopic fenestration necessary, injury of the urinary bladder; | Stable |
| 20 | Postrenal complication | Laparoscopic fenestration | None | Postrenal AKI II° |
| 21 | Progressive LC | Laparoscopic fenestration | None | Stable |
| 22 | Postrenal complication | Laparoscopic fenestration | None | AKI I° |
| 23 | Progressive LC | Laparoscopic fenestration | Injury of the ureter, infection | Stable |
| 24 | Progressive LC | Laparoscopic fenestration | None | Stable |
| 25 | Infection of LC | Open surgery | None | Stable |
| 26 | Progressive LC | Incision with drainage | None | Stable |
| 27 | Infection LC | Incision with drainage | Relapse – open surgery necessary | AKI I° |
| 28 | Progressive LC, compression of adjacent vessel | Incision with drainage | Relapse – laparoscopic fenestration necessary | AKI I° |
| 29 | Progressive LC | Laparoscopic fenestration | Injury of the ureter | Stable |
| 30 | Postrenal complication | Laparoscopic fenestration | None | AKI II° |
| 31 | Progressive LC | Laparoscopic fenestration | Injury of the ureter | Stable |
| 32 | Progressive LC | Laparoscopic fenestration | None | Stable |
| 33 | Progressive LC | Laparoscopic fenestration | None | Stable |
| 34 | Progressive LC | Laparoscopic fenestration | None | Stable |
| 35 | Progressive LC | Laparoscopic fenestration | None | Stable |
| 36 | Progressive LC | Laparoscopic fenestration | Relapse - 2nd laparoscopic fenestration necessary | Stable |
| 37 | Progressive LC, compression of adjacent vessel | Laparoscopic fenestration | None | Stable |
| 38 | Herniation | Laparoscopic fenestration | None | Stable |

| | | | | |
|---|---|---|---|---|
| 39 | Progressive LC, herniation | Laparoscopic fenestration | Relapse – open surgery necessary | Stable |
| 40 | Progressive LC | Laparoscopic fenestration | None | Stable |
| 41 | Progressive LC | Laparoscopic fenestration | Injury of the bladder | Stable |
| 42 | Progressive LC, herniation | Laparoscopic fenestration | None | Stable |
| 43 | Progressive LC, compression of adjacent vessel | Laparoscopic fenestration | None | Stable |
| 44 | Progressive LC, compression of adjacent vessel | Laparoscopic fenestration | 2x relapse, 2x laparoscopic fenestration | AKI I° |
| 45 | Progressive LC | Open surgery | None | Stable |
| 46 | Progressive LC | Laparoscopic fenestration | Relapse - 2nd laparoscopic fenestration necessary | Stable |
| 47 | Progressive LC | Incision with drainage | Relapse - laparoscopic fenestration necessary | Stable |
| 48 | Progressive LC, compression of adjacent vessel | Laparoscopic fenestration | None | Stable |
| 49 | Progressive LC | Laparoscopic fenestration | None | Stable |
| 50 | Progressive LC | Laparoscopic fenestration | Relapse - 2nd laparoscopic fenestration necessary | Stable |
| 51 | Progressive LC | Laparoscopic fenestration | Relapse - 2nd laparoscopic fenestration necessary | Stable |
| 52 | Progressive LC | Laparoscopic fenestration | None | Stable |
| 53 | Progressive LC, compression of adjacent vessel | Laparoscopic fenestration | None | Stable |
| 54 | Progressive LC | Laparoscopic fenestration | None | Stable |
| 55 | Progressive LC | Laparoscopic fenestration | None | Stable |
| 56 | Progressive LC, compression of adjacent vessel | Laparoscopic fenestration | None | Stable |
| 57 | Progressive LC | Laparoscopic fenestration | None | Stable |
| 58 | Progressive LC | Laparoscopic fenestration | Relapse - 2nd laparoscopic fenestration necessary | Stable |

| | | | | |
|---|---|---|---|---|
| 59 | Progressive LC | Laparoscopic fenestration | None | Stable |
| 60 | Progressive LC, postrenal complication | Laparoscopic fenestration | None | AKI II° |
| 61 | Progressive LC | Laparoscopic fenestration | None | Stable |
| 62 | Progressive LC | Laparoscopic fenestration | None | Stable |
| 63 | Progressive LC, compression of adjacent vessel | Laparoscopic fenestration | None | Stable |
| 64 | Progressive LC | Laparoscopic fenestration | None | Stable |
| 65 | Progressive LC | Laparoscopic fenestration | None | Stable |
| 66 | Progressive LC | Laparoscopic fenestration | None | Stable |
| 67 | Progressive LC | Open surgery | None | Stable |
| 68 | Progressive LC, compression of adjacent vessel | Laparoscopic fenestration | None | Stable |
| 69 | Progressive LC | Laparoscopic fenestration | Relapse - 2nd laparoscopic fenestration necessary | AKI I° |
| 70 | Progressive LC, compression of adjacent vessel | Laparoscopic fenestration | None | Stable |
| 71 | Progressive LC | Laparoscopic fenestration | None | Stable |
| 72 | Progressive LC | Incision with drainage | None | Stable |

LC= lymphocele; AKI=acute kidney injury graded by KDIGO 2012 guideline criteria [35]

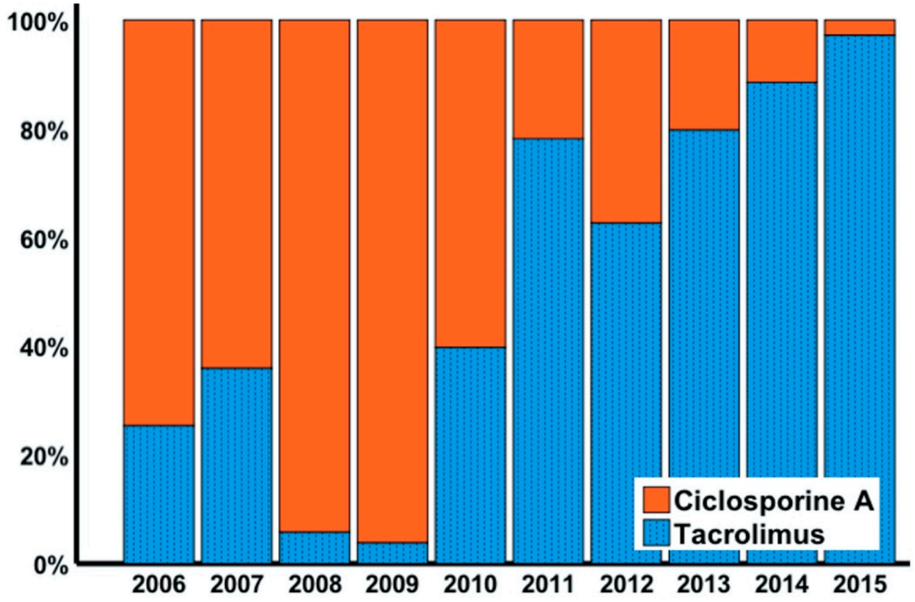


**Supplementary figure 1:** Proportion of Ciclosporin A & Tacrolimus treated patients by year.

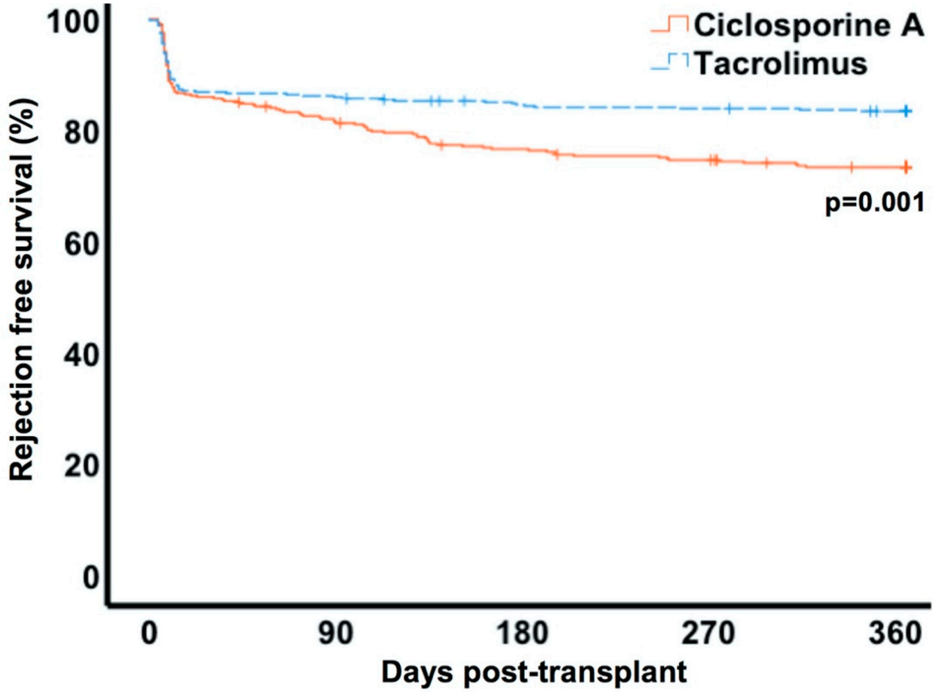


**Supplementary figure 2:** Rejection in the first year by calcineurin inhibitor use.

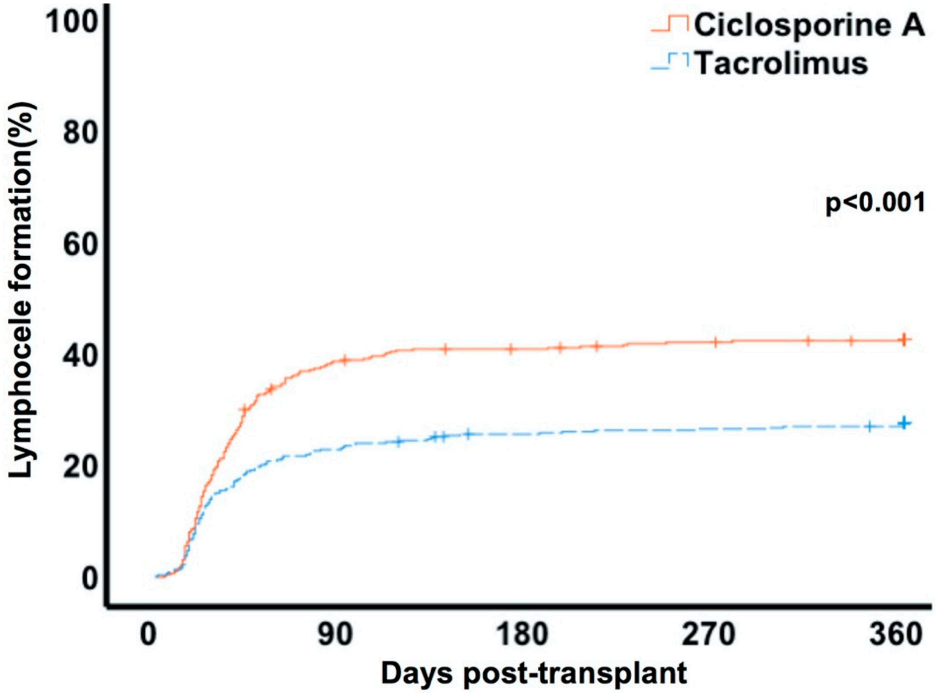


**Supplementary figure 3:** Lymphocele formation by calcineurin inhibitor use.
